# Carbohydrates – a scoping review for Nordic Nutrition Recommendations 2023

**DOI:** 10.29219/fnr.v67.10226

**Published:** 2023-11-10

**Authors:** Emily Sonestedt, Nina Cecilie Øverby

**Affiliations:** 1 Nutritional Epidemiology, Department of Clinical Sciences Malmö, Lund University, Malmö, Sweden; 2 Department of Nutrition and Public Health, Centre for Lifecourse Nutrition, University of Agder, Kristiansand, Norway

**Keywords:** carbohydrates, sugars, glycemic index, recommendations

## Abstract

**Background:**

Dietary carbohydrates are a major source of energy in the Nordic and Baltic countries. The health effects of different types of carbohydrates vary and there is a need to update the evidence regarding specific carbohydrates and their effects on health-related outcomes.

**Objective:**

The aim of this scoping review was to describe the evidence for the role of total carbohydrates (fiber excluded), glycemic index (GI) or glycemic load (GL) and added or free sugars for health-related outcomes as a basis for setting and updating dietary reference values for the Nordic Nutrition Recommendation (NNR) 2023.

**Method:**

We included evidence from several qualified systematic reviews (the World Cancer Research Fund, the European Food Safety Authority, the World Health Organization, the United States Dietary Guidelines Advisory Committee, the United Kingdom Scientific Advisory Committee on Nutrition) identified by the NNR project in line with the protocol description.

**Results:**

There is limited evidence that total carbohydrate intake (fiber excluded) outside of the current recommended range of 45–60% of energy is associated with health-related outcomes. There were no consistent benefits on clinical outcomes when changing the GI of a diet. High intake of dietary sugars is well known to be associated with dental caries. There was evidence from randomized control trials on surrogate disease endpoints, for a positive and causal relationship between the intake of added and free sugars and risk of some chronic metabolic diseases with moderate level of certainty for obesity and dyslipidaemia. The level of certainty was high for an association between high intake of sugar-sweetened beverages and risk of several chronic metabolic diseases.

**Conclusion:**

While there is limited evidence that total carbohydrates and GI and GL of the diet are related to health outcomes, the evidence suggests that high intakes of added and free sugars are related to detrimental health effects. In addition, with increasing intake of added and free sugars, there is less room for healthy foods and micronutrients, which is especially important for those with low energy intake, such as children.

## Popular scientific summary

- Intake of carbohydrates in the Nordic and Baltic countries varies from 46 to 54% of total energy among children and from 37 to 47 E% in adults.
- Evidence for health effects of total carbohydrate intakes outside the recommended range of 45–60% of energy is limited.
- High intake of added and free sugars is associated with risk obesity, dyslipidaemia and dental caries.
- There is strong evidence relating high intakes of sugar-sweetened beverages with risk of chronic metabolic diseases.

The chemical classification of carbohydrates is usually based on molecular size and monomeric composition. The four main groups of carbohydrates are monosaccharides (1 monomer), disaccharides (2 monomers), oligosaccharides (3–9 monomers) and polysaccharides (10 or more monomers). The term ‘sugars’ (total sugar) covers monosaccharides and disaccharides. The sugars found in the highest amount in food are glucose, fructose and galactose (monosaccharides) and sucrose, lactose and trehalose (disaccharides). There are two main classes of polysaccharides, starch and non-starch polysaccharides. Starch is a homopolymer of glucose and comes in two main forms, amylose (basically unbranched) and amylopectin (highly branched) (1). Non-starch polysaccharides include, for example, cellulose and pectin. Other types of carbohydrates are sugar alcohols (polyols) such as sorbitol, xylitol and mannitol and organic acids such as lactic acid and citric acids (1). Nutritionally, carbohydrates can be divided into two broad categories. The first includes those that are digested and absorbed in the human small intestine providing carbohydrates to body cells, that is, glycemic carbohydrates. The second includes the non-digestible (unavailable) carbohydrates, that is, dietary fiber, and is described in a separate scoping review in the journal.

In the literature, various terms are used to differentiate between sugars naturally occurring in foods, that is, ‘intrinsic’ sugars, and sugars and sugar preparations added to foods, that is, ‘added’ or ‘extrinsic’ sugars (2). In the Nordic Nutrition Recommendations (NNR) 2012, the term ‘added sugars’ referred to refined sugars such as sucrose, fructose, glucose, starch hydrolysates (glucose syrup, high-fructose syrup) and other isolated sugar preparations used as such or added during food preparation and manufacturing (1). A similar definition is used in the US dietary guidelines (3). In addition, the term ‘free sugars’ is used in guidelines, for example, from the WHO (4) and the United Kingdom (5) and in the European Food Safety Authority (EFSA) scientific opinion on tolerable upper level for dietary sugars (6). Free sugars include added sugars plus sugars naturally present in honey, syrups, fruit juices and fruit juice concentrates (6). In NNR 2012, the recommendation was to limit the intake of added, refined sugars below 10 E% for adults and children above 6 months of age. The basis for this recommendation was the importance to ensure adequate intakes of micronutrients and dietary fiber (nutrient density) as well as supporting a healthy dietary pattern and reduce risk of dental caries. Another important foundation for the recommendation was the evidence on sugar-sweetened drinks’ association with an increased risk of type 2 diabetes (7) and excess weight gain. The recommended interval for total carbohydrates was in NNR 2012 set to 45–60 E% for adults and children from 6 months of age. This range was primarily based on that typical ranges of total carbohydrate intakes in studies on dietary patterns associated with reduced risk of chronic diseases among adults were within 45–60 E%. The recommendation also stated that whole grain cereals, whole fruit, berries, vegetables and pulses should be the major sources of dietary carbohydrates. There was not enough evidence for including glycemic index (GI) or glycemic load (GL), that is, the estimated blood glucose increasing effect of a diet, as a recommendation (1). The aim of this scoping review was to describe the totality of evidence for the role of total carbohydrates (fiber excluded), GI or GL, and added and free sugar for health-related outcomes as a basis for setting and updating dietary reference values (Box 1).

Box 1. *The review process of scoping reviews in the NNR2023 project*

- This paper is one of many scoping reviews commissioned as part of the Nordic Nutrition Recommendations 2023 (NNR2023) project (8).
- The papers are included in the extended NNR2023 report but, for transparency, these scoping reviews are also published in Food and Nutrition Research.
- The scoping reviews have been peer reviewed by unrelated experts in the research field according to the standard procedures of the journal.
- The scoping reviews have also been subjected to public consultations (see report to be published by the NNR2023 project).
- The NNR2023 committee has served as the editorial board.
- While these papers are a main fundament, the NNR2023 committee has the sole responsibility for setting dietary reference values in the NNR2023 project.

## Methods

This scoping review follows the protocol developed within the NNR2023 project (8). The sources of evidence used in this scoping review follow the eligibility criteria described in the paper ‘The Nordic Nutrition Recommendations 2022 – Principles and Methodologies’ (9). None of the de novo systematic reviews conducted by NNR investigated carbohydrate or sugar intake (10). Several qualified systematic reviews commissioned by international organizations and health authorities were identified by the NNR committee in 2021 for total carbohydrates, GI, GL or added or free sugars (11). We examined qualified systematic reviews published after 2012, as well as a systematic review by EFSA published in 2022. These are listed by years published below:

- **The European Food Safety Authority (EFSA) (2022)** was asked to deliver a scientific opinion on the tolerable upper intake level for dietary sugars, that is, maximum level of chronic daily intake of sugar judged to be unlikely to pose a risk of adverse health effects. They performed systematic reviews of the relationship between intake of total and added or free sugars and chronic metabolic diseases, pregnancy-related endpoints and dental caries. The search was conducted in July 2018 and updated in August 2020 (metabolic diseases) or October 2020 (dental caries) (6). This is an update of the Scientific Opinion on Dietary Reference Values for carbohydrates published 2010 (12).

- **The 2020 Dietary Guidelines Advisory Committee (DGAC)** conducted systematic reviews to form the basis for the **Dietary guidelines for Americans 2020** (3). Results from the following subcommittees are used in this paper: dietary patterns subcommittee (macronutrient composition), the beverages and sugars subcommittee and the birth to 24 months subcommittee. Specific protocols are provided by the DGAC on each systematic review and most searches include literature up to September/October 2019.

- **Reynolds et al. (2019) commissioned by the WHO** performed a series of systematic reviews and meta-analyses of prospective studies published until April 2017 and randomized controlled trials (RCTs) published until February 2018, which reported on indicators of carbohydrate quality (i.e. whole grains, fiber, and GI and GL) and non-communicable disease incidence, mortality and risk factors that have been measured in an appreciable number of studies. Dietary GI and GL were the only relevant exposures for the present scoping review. They used Grading of Recommendations Assessment, Development and Evaluation (GRADE) to assess quality of evidence (13).

**- The World Health Organization (WHO) Guideline: sugars intake for adults and children (2015)**, aiming to provide recommendations on intake of free sugars to reduce the risk of non-communicable diseases in adults and children, used the WHO procedures and GRADE methodology to assess the quality of evidence (4). Two systematic reviews were performed assessing the effects of increasing or decreasing intake of free sugars on excess weight (14) and dental caries (15). Both reviews include studies up to or through November 2011.

- **UK Scientific Advisory Committee on Nutrition (SACN) Carbohydrates and Health Report (2015)** presents systematic reviews of the evidence on carbohydrates and cardiometabolic, colorectal cancer and oral health. Literature published from 1990 up to and including December 2009, November 2010 and January 2011, respectively, and an updated search in June 2012. Evidence from prospective cohort studies and RCTs was considered for this report. Evidence was assessed using the SACN Framework for the Evaluation of Evidence and graded according to a system developed specifically for the review (5).

- **World Cancer Research Fund (WCRF)** and the American Institute for Cancer Research summarize continuously the evidence on how diet affects the risk of developing cancer. Systematic literature reviews for each cancer type have been performed and the evidence has been graded. The exposures relevant for this paper are total carbohydrates, GI and GL, and fructose (16).

To summarize the evidence on the association between consumption of added and free sugars and diet quality and micronutrient dilution, we conducted a search on Web of Science on October 25, 2022, among articles that have cited the systematic review ‘Association between intake of total versus added sugar on diet quality: a systematic review’ published in 2015 by Louis et al. Among the 57 identified articles, 9 were included.

## Physiology

### Digestion, absorption and metabolism

To be absorbed in the small intestine, carbohydrates must be broken down into monosaccharides. The enzymatic degradation of starch begins by the action of salivary amylase in the oral cavity and is continued in the small intestine by pancreatic amylase. The degradation products – mainly maltose and maltotriose – and sucrose, lactose and other digestible di- and oligosaccharides are further hydrolyzed to glucose by enzymes (e.g. alpha glycosidase, sucrase, maltase and lactase) that are bound to the brush border membrane of the enterocytes. Glucose and galactose are absorbed efficiently by a secondary active carrier coupled with sodium (SGLT1), whereas fructose is absorbed by facilitated diffusion that does not involve sodium co-transport (GLUT5). In addition, all monosaccharides are absorbed by passive diffusion with GLUT2 (1, 6).

After absorption, the monosaccharides are transported via the portal vein to the liver. Most of the glucose is transported to the systemic circulation leading to the increase of blood glucose concentration. Glucose can also be stored as glycogen in the liver and in the muscles. The storage capacity is limited to around 500 g, of which 300–400 g can be stored in the muscles. Liver glycogen is used to maintain normal blood glucose concentrations between meals, and muscle glycogen is used primarily as a source of energy within the muscles. Fructose is converted to lactate or glucose in the liver or, similar to galactose, to glycogen and is therefore found in very small amounts in the systemic circulation. Thus, the glycemic carbohydrates reach the peripheral circulation mainly as glucose. The metabolism of fructose favors lipogenesis more than glucose (1).

The blood glucose concentration is tightly controlled in healthy individuals via insulin, the main blood glucose-lowering hormone, and glucagon, the main blood glucose-raising hormone. Insulin is secreted in response to the elevated blood glucose concentration after a meal. In addition, vagal signals, gastrointestinal hormones (incretins) and certain non-carbohydrate food components – especially amino acids – contribute to the stimulation of insulin secretion. The blood glucose concentration can be lowered mainly through (1) the peripheral glucose uptake, which is dependent on both insulin production by the pancreas and the level of peripheral insulin sensitivity or resistance, (2) increased glycogen synthesis and (3) decreased gluconeogenesis (formation of glucose) in the liver (1). In diabetes, the glucose regulation is impaired and blood glucose is therefore permanently increased. In type 1 diabetes, the insulin-producing beta cells in pancreas are damaged by the immune system. In type 2 diabetes, the tissues are insulin resistant and therefore cannot absorb glucose and/or insufficient insulin production by the pancreas.

### Glycemic index and glycemic load

The concept of GI was introduced by Jenkins and coworkers in 1981 (17) to rank foods in a standardized way with regard to their effects on blood glucose levels after a meal. GI is defined as the incremental area under the blood glucose response curve after a 50 g carbohydrate portion of a test food expressed as a percentage of the response to the same amount of carbohydrate from a standard food (either glucose or white bread) taken by the same subject. The factors that determine the GI of a carbohydrate are generally unrelated to the molecular size of the carbohydrate. For instance, both fructose and sucrose have a lower GI than white bread (18). Starchy foods, on the other hand, can have a low, intermediate or high GI depending on the composition (amylose/amylopectin ratio), amount of resistant starch and physical/chemical state. Physical barriers such as intact cereal grains, the cellular structures in leguminous seeds, parboiled rice and whole fruits, low degree of food processing and the protein network in pasta products are examples of food-related factors lowering the GI (19). The concept of GL was introduced in 1997 by Harvard epidemiologists to quantify the glycemic effect of a portion of food (20). GL is defined as the amount of glycemic carbohydrate in a food multiplied by the GI of the food divided by 100. The glycemic response to a meal can also be influenced by the protein and fat content as well as by the size of the meal and the amount of drink taken with the food.

## Dietary intake in Nordic and Baltic countries

Carbohydrate intakes in different age groups of the population in the Nordic and Baltic countries using data from national dietary surveys are summarized in Table 1 (21). Mean energy percentage from carbohydrates (E%) ranges from 46 to 54 E% among children aged 2–18 years and from 37 to 47 E% in adults.

**Table 1:** Intake of available carbohydrates (E%) in different age groups across Nordic and Baltic countries

Country	Age (years)	Carbohydrates, E%			
		Men		Women	
		*N*	Mean	*N*	Mean
Denmark	4–5	66	48	64	48
	6–9	150	49	141	49
	10–13	134	49	135	49
	14–17	117	46	123	48
	18–75	1,464	41	1,552	43
Finland	3–4	239	49	228	48
	5–6	184	49	164	50
	18–74	780	41	875	43
Iceland	18–80	632	41	680	43
Norway	9*	295	50	341	51
	13*	332	49	355	50
	18–70	862	45	925	46
Sweden	12	490	47	559	47
	15	476	46	574	47
	18	423	44	577	46
	18–80	792	43	1,005	44
Estonia	2–5	277	54	302	54
	6–9	168	52	179	52
	10–13	93	50	89	53
	14–17	80	51	117	50
	18–74	907	45	1,806	48
Latvia	19–64	470	39	541	39
Lithuania	19–64	1,044	42	1,469	46

* Data were collected from children attending 4th and 8th grade.

Data were obtained from NNR report using data from the most recent national dietary surveys in each of the Nordic and Baltic countries (21).

Data on intakes of total, free and added sugars are retrieved from the EFSA report published in 2022, with data from Denmark, Estonia, Finland, Latvia and Sweden (6), which is shown in Table 2. These were the most recent data presented collectively, not all data might be up to date as countries regularly conduct national dietary surveys. Estimates were obtained using data from the EFSA Comprehensive Food Consumption Database in combination with the food composition databases for total, free and added sugars. For infants, mean intake of total sugars ranges from 24 E% to 35 E%, while mean intake of free sugars and added sugars ranges from 1 E% in Estonia to 11 E% in Finland. Contributions for free sugars and added sugars differ between countries, but ‘sugars and confectionery (i.e. sugar, honey, syrups, confectionery and water-based sweet desserts)’, sweetened milk and dairy and baby foods and processed fruit (free sugars) are important sources. For toddlers, mean intakes of total sugars range from 24 E% to 29 E%. Free sugars range from 9 E% to 11 E%, while added sugars range from 7 E% to 9 E%. Dietary sources of free sugars are primarily sugars and confectionery, juices and fine bakery. For added sugars, fruit juices are not a major source.

**Table 2:** Intakes of total, free and added sugars (E%) in different age groups across Nordic and Baltic countries^1^

Age group	Country	Survey	*N*	Total sugar		Free sugar		Added sugar	
				Mean	P95	Mean	P95	Mean	P95
Infants	Denmark	IAT 2006–07	826	24	36	3	9	3	8
	Estonia	DIET-2014-EST-C	493	35	41	1	6	1	5
	Finland	DIPP	500	34	50	11	31	11	30
	Latvia	LATVIA_2014	119	34	50	5	12	4	11
Toddlers	Denmark	IAT 2006–07	917	24	33	9	18	7	14
	Estonia	DIET-2014-EST-C	268	27	42	11	23	9	18
	Finland	DIPP 2001–2009	500	29	39	9	19	8	16
	Latvia	LATVIA_2014	242	24	37	9	19	8	15
Children	Denmark^2^	DANSDA 2005–2008	298	37	50	21	35	18	29
	Estonia	DIET-2014-EST-C	104	27	40	13	23	11	20
	Finland	DIPP 2001–2009	750	28	37	15	26	10	17
	Latvia	LATVIA_2014	782	24	34	13	22	11	18
	Sweden	NFA 2018	1,473	27	37	16	26	14	24
Adolescents 10–14 years	Denmark^2^	DANSDA 2005–2008	233	36	55	22	37	19	34
	Estonia	DIET_2014-EST-A	107	24	37	13	24	11	22
	Finland	NWSSP07_08	198	26	41	15	30	10	21
	Latvia	LATVIA_2014	338	22	32	12	21	10	18
	Sweden	NFA 2018	1,018	24	35	15	27	13	24
Adolescents 14–18 years	Denmark^2^	DANSDA 2005–2008	144	36	54	21	38	18	32
	Estonia	DIET_2014-EST-A	193	23	38	12	23	10	19
	Finland	NWSSP07_08	108	26	37	15	30	10	20
	Latvia	LATVIA_2014	282	20	30	11	18	9	16
Adults	Denmark^2^	DANSDA 2005–2008	1,739	32	52	17	35	14	30
	Estonia	DIET-2014-EST-A	2,124	21	35	9	19	7	16
	Finland	FINDIET2012	1,295	22	38	11	24	8	19
	Latvia	LATVIA_2014	1,080	19	33	10	21	9	18
	Sweden	RIKSMATEN 2010	1,430	18	29	9	19	8	16
Elderly	Denmark^2^	DANSDA 2005–2008	286	31	46	15	28	12	26
	Estonia	DIET-2014-EST-A	525	21	34	9	19	6	15
	Finland	FINDIET2012	413	23	35	9	20	7	15
	Latvia	LATVIA_2014	310	19	29	9	18	8	17
	Sweden	RIKSMATEN 2010	367	19	30	9	18	7	15

1 Data were obtained from the EFSA report supplementary information (Annex D, Table 1) that have used data from respective country’s national dietary surveys (37).

2 Data on energy intake of the corresponding survey were not considered in the intake assessment.

For children and adolescents, intake of total sugars ranges from 20 E% to 28 E% in most countries and groups, while Denmark has a higher intake (36–37 E%). Intakes of free sugars range from 11 E% to 16 E%, with 22 E% in Denmark, while intakes of added sugars range from 10 E% to 19 E% in Denmark. According to the EFSA report, there are some uncertainties in the Danish data ‘as energy intake was not considered in the intake assessment’. For children and adolescents, the major sources for free sugars are fruit and vegetable juices, sugars and confectionery, fine bakery and sugar-sweetened beverages. Sources for added sugars are sugars and confectionery, sugar-sweetened beverages and fine bakery wares.

Intakes among adults and elderly range for total sugars from 18 E% to 32 E%, for free sugars from 9 E% to 17 E% and for added sugars from 8 E% to 14 E%. Intakes in lactating and pregnant women are reported from Estonia and Latvia, and intakes range from 20 E% to 23 E% for total sugars, 10 E% for free sugars and 8 E% to 9 E% for added sugars. For adults and elderly, sugars and confectionery and juices are the main sources for free sugars intake. For added sugars, sugars and confectionery, fine bakery products and sugar-sweetened beverages are major sources.

## Carbohydrates, glycemic index, glycemic load and added or free sugars and health outcomes

### Total carbohydrates

#### Obesity

The US review reported that there is no evidence available to determine a relationship between diets based on macronutrient distribution consumed during childhood and growth, body size, body composition and risk of overweight/obesity (3). The SACN review reported no association in children between total carbohydrate intake and body mass index and body fatness (limited evidence) (5). Further, in adults, the US review reported that insufficient evidence is available to determine the relationship between macronutrient distributions with proportions of energy falling outside of the acceptable macronutrient distribution range for at least one macronutrient (i.e. 45–60 E% for carbohydrates, 25–35 E% for fat and 10–35 E% for protein) and growth, body size, body composition and risk of overweight/obesity. The insufficient evidence was due to methodological limitations and inconsistent results. The limitations are, for example, not adjusting for relevant confounders and diet assessment methods varying in validity and reliability (3). The SACN review reported no effect of higher carbohydrate diet on fat free mass (moderate evidence) and waist to hip ratio (limited evidence) in adults (5).

#### Cardiovascular diseases

The US review reported that no evidence was available to determine the relationship between diets based on macronutrient distribution consumed by children or adolescents and concurrent or future development of cardiovascular disease (3). Limited evidence suggests that non-energy restricted diets based solely on macronutrient distribution are neither beneficial nor detrimental regarding risk of cardiovascular disease in adults, primarily among those at high risk, such as those with overweight, obesity or features of metabolic syndrome (3). The SACN review reported that no significant association was observed in any of the included studies between total carbohydrate intake and cardiovascular events (moderate evidence) (5).

#### Type 2 diabetes

The US review stated that there is no evidence available to determine a relationship between diets based on macronutrient composition consumed during childhood and risk of type 2 diabetes. In addition, insufficient evidence is available to determine the relationship between macronutrient composition and risk of type 2 diabetes in adults, due to methodological limitations and inconsistent results. Methodological issues were that studies did not directly test differences in macronutrient proportions in the context of a constant dietary pattern and that the gradient between macronutrient proportions compared within and across studies varied (3). The SACN report reviewed evidence on total carbohydrates and type 2 diabetes and found no significant association between total carbohydrate intake and incidence of type 2 diabetes (5).

#### Cancers

The WCRF review, including five studies, showed an increased risk of endometrial cancer with an increase in carbohydrate intake (16). The SACN review reported no association between total carbohydrate and colorectal cancer incidence (adequate evidence) (5).

#### Mortality

The US review presented 28 articles from prospective cohort studies examining the relationship between diets based on macronutrient distribution and mortality. Diets with carbohydrates composition between 45 and 60 E% compared to outside this range tended to be associated with reduced all-cause mortality, particularly when the diets examined were of higher quality (i.e. emphasizing vegetables, fruits, nuts, whole grains, legumes, fish and/or lean meat or poultry) (3). In the studies where food quality was not assessed, the results were inconsistent. The US review report concluded that there is insufficient evidence available to determine the relationship between diets based on macronutrient distribution and all-cause mortality (3).

#### Cardiometabolic risk markers

The SACN review reported no effect of higher carbohydrate, lower fat diets on systolic blood pressure or diastolic blood pressure (5). A diet higher in carbohydrate and lower in fat may decrease fasting total cholesterol concentration compared to lower carbohydrate, higher fat diets, but it was not possible to exclude confounding by other variables, for example, a concomitant reduction in saturated fat intake and/or weight loss. No effect was found for higher carbohydrate, lower fat diets and fasting low density lipoprotein (LDL)- or high densitiy lipoprotein (HDL)-cholesterol concentration (5).

### Glycemic index and glycemic load

In the systematic review by Reynolds et al., diet with lower overall GI appears to be associated with reduced risk of stroke mortality (three studies) and type 2 diabetes (14 studies). However, the evidence was graded as low for stroke mortality and very low for type 2 diabetes. In addition, there is low evidence that diets characterized by lower GI have an effect on coronary heart disease incidence compared to diets with higher GI and very low evidence for an effect on all-cause mortality, coronary heart disease mortality, cancer mortality, stroke incidence and colorectal cancer incidence (13).

The SACN review reported that there is an association between diets characterized by higher GI and GL and higher incidence of type 2 diabetes but confounding cannot be excluded (adequate evidence). The SACN review concluded that there is limited evidence on an association between higher GI and GL and incidence of cardiovascular disease events (5).

The WCRF review concluded that diets characterized by higher GL are probably a cause of endometrial cancer based on results from six cohort studies (16). Regarding other types of cancer, too limited information was available to draw a conclusion on the association between GL and cancer in the pancreas, liver, colorectal and breast (16). The SACN review reported no association between GI or GL and incidence of colorectal cancer, which was the only cancer investigated (5).

In the systematic review by Reynolds et al., they reported effects from RCTs of diets characterized by lower compared to higher GI on change in body weight, glucose homeostasis, cholesterol levels and blood pressure. They excluded trials that involved only people with diabetes or marked hyperlipidemia. Overall, the results from intervention trials showed no consistent benefits on clinical outcomes when changing the GI of a diet (13). The SACN review reported an effect of GI on total and LDL-cholesterol concentrations (moderate evidence). A higher GI diet may result in less of a reduction in fasting total cholesterol and in fasting LDL-cholesterol, respectively, compared with a lower GI diet. Further, they reported no effect of GI on fasting HDL-cholesterol and triacylglycerol concentration (moderate evidence) (5). Interestingly, none of the same studies were included in reviews by Reynolds et al. and SACN. While Reynolds et al. excluded weight loss trials, the SACN review included such trials. This may explain the different findings between the two systematic reviews.

### Total sugars

In the EFSA review, the available body of evidence from prospective cohorts does not support a positive and causal relationship between the intake of total sugars in isocaloric exchange with other macronutrients and any of these chronic metabolic diseases (obesity, non-alcoholic fatty liver disease, type 2 diabetes, dyslipidemia, hypertension and cardiovascular disease) (6). There were no intervention studies on total sugars and pregnancy outcomes, and only one cohort study included data on total sugar. Based on the limited number of studies, the EFSA Panel concludes that the available evidence does not support a positive relationship between the intake of total sugars in isocaloric exchange with other macronutrients and risk of gestational diabetes (GDM). The EFSA Panel concluded that the available evidence cannot be used to conclude on a positive and causal relationship between the intake of total sugars and risk of adverse effects on birthweight. The SACN review reported no association between sugar consumption and incidence of coronary events in adults (moderate evidence) (5), incidence of type 2 diabetes (limited evidence), no effect of sugar consumption on fasting blood glucose concentration (limited evidence) and insulin concentration (limited evidence). Further, they found no association between sucrose and incidence of type 2 diabetes (limited evidence), and between glucose, fructose or lactose and type 2 diabetes (limited evidence) (5). The SACN review reported that there is no effect demonstrated in the proportion of sugar (E%) on systolic blood pressure (limited evidence), fasting total cholesterol concentration (limited evidence), fasting LDL-cholesterol concentration (limited evidence), fasting HDL-cholesterol concentration (limited evidence) and fasting triacylglycerol concentration (limited evidence) (5). Lastly, the SACN review also reported an association between sugar intake and oral health (moderate evidence) (5).

### Fructose

In the EFSA review, there was evidence from prospective cohort studies for a positive and causal relationship between the intake of fructose and risk of gout (moderate certainty; >50–75% probability) and risk of cardiovascular diseases (low certainty; >15–50% probability), although the external validity of the findings for European populations is unclear. In the eligible RCTs, the effects of fructose and glucose on body weight, liver fat, measures of glucose tolerance, blood lipids and blood pressure did not differ, whereas fructose appeared to increase hepatic insulin resistance and uric acid levels more than equivalent amounts of glucose (6). The WCRF review concluded that there is an increased risk of pancreatic cancer with higher intake of fructose (limited suggestive evidence). Although there is ample evidence, which is generally consistent and there is some evidence for a dose-response relationship, fructose comes from many sources making the evidence difficult to interpret. Therefore, the evidence suggesting that foods and beverages containing fructose are a cause of pancreatic cancer is limited (16). For other related exposures (total carbohydrate, sucrose and soft drinks), there were no clear associations with pancreatic cancer risk (evidence is too limited to draw conclusion) (16). The SACN report found no association between fructose and type 2 diabetes (limited evidence) (5).

### Added and free sugars

The EFSA review has investigated added sugars, sucrose (as a proxy for added sugars) and free sugars combined to draw conclusions in relation to the endpoints of interest. This is owing to the low number of studies available per each exposure and endpoint and the fact that intakes of added and free sugars widely overlap. Therefore, the health effects of added versus free sugars could not be compared. Below we give an overview of evidence from different bodies where some present added sugars and some free sugars, while EFSA has combined these.

#### Obesity

The EFSA review found that there is evidence from RCTs for a positive and causal relationship between the intake of added and free sugars ad libitum and risk of obesity (moderate level of certainty). Using data from 11 RCTs, body weight was on average 1.15 kg (95% confidence interval [CI]: 0.53, 1.77) higher in the group receiving high sugar diet compared to the low sugar diet. Between-arm differences in added and free sugars intake were between 6 and 24 E%. Most RCTs were in adults with overweight or obesity, and two of the studies were in children and adolescents. The available body of evidence from prospective cohorts does not show a positive relationship between the intake of added and free sugars and obesity in isocaloric exchange with other macronutrients (6). The WHO review found moderate evidence using data from RCTs suggesting an association between reduction of free sugars intake and reduced body weight in adults. Also in adults, an increased intake of free sugars was associated with a comparable increase in body weight. Summing up evidence for children, the WHO reported that the RCTs in children comprising interventions with recommendations to reduce sugar-sweetened foods and beverages were characterized with low compliance and showed no overall change in body weight (low quality of evidence). However, meta-analysis of prospective cohort studies found that children with highest intakes of sugar-sweetened beverages had a greater likelihood of being overweight or obese than children with the lowest intakes (moderate quality of evidence) (4). In addition, the US dietary guideline reported evidence suggesting that sugar-sweetened beverage consumption during the complementary feeding period is associated with increased risk of obesity in childhood (limited evidence), but is not associated with other measures of growth, size and body composition (3). Further, limited evidence showed a positive association between juice intake and infant weight for length and child body mass index z-scores.

#### Cardiovascular disease

In the EFSA review, no RCTs on added/free sugars intake and cardiovascular disease endpoints (i.e. incidence and mortality of CVD) were available, and only three prospective cohort studies were included in the EFSA report on the relationship between added sugar, free sugar or sucrose and cardiovascular disease or cardiovascular mortality. The EFSA Panel concluded that the available data cannot be used to conclude on a positive and causal relationship between the intake of added and free sugars, in isocaloric exchange with other macronutrients and risk of CVDs (6). However, the EFSA reported a high level of certainty for a positive and causal relationship between the intake of sugar-sweetened beverages and risk of CVD. The relationship was observed for sugar-sweetened beverages when not keeping total energy intake constant (i.e. not adjusting for total energy intake) (6). In the US review, they reported limited evidence from prospective cohort studies (primarily based on sugar-sweetened beverages) to suggest that higher consumption of added sugars in adulthood is associated with increased risk of cardiovascular disease mortality (3). The review reported from three large prospective studies examining total added sugars and CVD-related mortality. Two found no significant association, while one found an association before adjustment for adiposity. Five additional prospective studies examined the relationship between added sugars in the form of sugar-sweetened beverages and CVD-related mortality, four found no effect, while the fifth with better design found significant deleterious effect in both men and women measuring intake multiple times. Insufficient evidence was available to determine the relationship between consumption of added sugars and risk of cardiovascular disease in children (3). In addition, insufficient evidence is available to determine the relationship between added sugars intake in adulthood and risk of stroke, ischemic cardiovascular disease, peripheral artery disease and heart failure (3).

#### Type 2 diabetes

The level of certainty for a positive and causal relationship between the intake of added and free sugars and risk of type 2 diabetes is low, according to the EFSA. There were no RCTs on the incidence of type 2 diabetes, so surrogate measures (glucose tolerance, insulin sensitivity) were reported. Using data from 17 RCTs, fasting glucose was on average 1.94 mg/L higher in the high added or free sugar arm relative to the low added or free sugar arm. Results on insulin sensitivity were mixed. The available evidence from prospective studies could not modify the level of certainty in this conclusion (6). However, the EFSA found there is strong evidence from prospective cohort studies for a positive relationship between the intake of sugar-sweetened beverages and risk of type 2 diabetes (high certainty) and evidence from RCTs supports this relation (low certainty) (6).

#### Cancer

None of the qualified systematic reviews presented evidence on intake of added or free sugars and cancer risk. According to the SACN review, there was no association between sugar-sweetened beverages consumption and colon cancer (adequate evidence) (5).

#### Cardiometabolic risk markers

In the EFSA review, there is evidence from RCTs for a positive and causal relationship between the intake of added and free sugars and risk of dyslipidemia (moderate level of certainty). Based on data from 24 RCTs, total cholesterol and fasting triglycerides were higher in the high versus the low added or free sugar arm. LDL-cholesterol was also higher based on 17 RCTs; however, HDL-cholesterol was minimally affected by the interventions based on 20 RCTs. All studies were conducted in adults and six RCTs were in healthy subjects and the remaining in selected subgroups (e.g. subjects with overweight, obesity or hypertriglyceridemia). The effect of high versus low sugar intake was of bigger magnitude for all blood lipid variables when the analysis was restricted to studies conducted under neutral energy balance in isocaloric exchange with starch. The available body of evidence from the three identified prospective cohort studies could not be used to modify the level of certainty in this conclusion (6). The EFSA also reported evidence for a positive association between intake of sugar-sweetened beverages and dyslipidemia (low level of certainty). Of other cardiovascular risk factors, the EFSA reported a positive relationship between the intake of added and free sugars ad libitum and isocaloric exchange with starch and risk of hypertension (very low level of certainty). Further, the EFSA reported that there is evidence from prospective studies for a positive association between sugar-sweetened beverages and risk of hypertension (high level of certainty), with evidence from RCTs (very low certainty) supporting the relationship (6). According to the US review, there is insufficient evidence available to determine the relationship between added sugars intake in adulthood and cardiovascular disease risk profile (3) and insufficient evidence to determine the relationship in children.

#### Pregnancy and neonatal health

The EFSA Panel included some evidence of sugar-sweetened beverages and pregnancy and neonatal outcomes. Based on data from two observational studies, the EFSA states that there is evidence for a positive and causal relationship between the intake of sugar-sweetened beverages and risk of GDM (low level of certainty) (6). The EFSA Panel also concluded that there is evidence from observational studies for a positive and causal relationship between the intake of sugar-sweetened beverages and risk of adverse effects on birthweight, although there is a very low level of certainty (6).

#### Dental caries

It is well established that dietary sugars are involved in the development of dental caries at all ages (22). However, the EFSA review stated that the available body of evidence does not allow conclusions on the shape of the relationship between the intake of dietary sugars and risk of dental caries for any age group, or to identify a level of sugars intake at which the risk of dental caries is not increased (6). The WHO report stated that there is moderate evidence for positive association between the level of free sugars intake and dental caries in both adults and children. The evidence suggests higher rates of dental caries when the level of free sugars intake is more than 10% of total energy intake compared with being less than 10% of total energy intake. Further, the WHO review reported from three national population studies that lower levels of dental caries development were observed when per capita sugars intake was less than 10 kg/person/year (approx. 5% of total energy). Further, a positive log-linear dose-response relationship between free sugars intake and dental caries was observed across all studies in the WHO review, at free sugars intakes well below 10 kg/person/year. Quality of evidence from cohort studies was considered to be moderate, while evidence from the national population studies was considered to be very low (4).

#### Micronutrient dilution

In addition to health issues, one of the main reasons for limiting intake of added and free sugars is the fact that added sugar only provides energy and not nutrients. This means that with increasing intake of added and free sugars, there is less room for healthy foods and essential nutrients, a phenomenon known as micronutrient dilution. This is especially important for those with low energy intake, such as children. In a systematic review, published in 2015, a negative association between added sugar and micronutrient intake was found, while no such association was found for total sugar intake (23). We performed a literature search on studies that cited this systematic review and identified nine studies (cross-sectional) addressing the child, adolescent, adult and elderly population, from Finland, the United Kingdom, Australia, Japan and Saudi Arabia. All studies reported negative associations between added or free sugars intake and either micronutrient intake or diet quality (24–33). In addition, a study from the Swedish population not identified in this search showed similar results (34). The 2020 American Dietary Guidelines Advisory Committee has evaluated the scientific evidence for the potential health impacts of added sugars intake, along with the findings from model-based estimations of energy available in the dietary pattern after meeting nutrient requirements. Based on these, they suggest that less than 6 E% from added sugars is more consistent with a dietary pattern that is nutritionally adequate while avoiding excess energy intake from added sugars than is a pattern with less than 10 E% from added sugars. The combinations of foods needed to achieve recommended intakes of key nutrients for ages 6–24 months leave virtually no remaining dietary energy for added sugars, apart from the very small amounts (less than 3 g per day) already inherent in the foods used in modeling. For the NNR we suggest that such modeling is done on Nordic and Baltic studies to have evidence on how higher intake of added and free sugars dilutes the diet quality. This is in line with the EFSA Panel noting that the lowest amounts of added and free sugars that are compatible with a nutritionally adequate diet may vary across population groups and countries.

## Requirement and recommended intakes

### Summary of main results

In conclusion, there is not enough evidence that carbohydrate intakes outside the current recommended range of 45–60 E% are related to health effects. However, diets with proportions of carbohydrates within this range tend to be associated with reduced all-cause mortality among adults, particularly when the diets examined were of higher quality. Thus, the evidence indicates that total carbohydrate intake appears to be neither detrimental nor beneficial to cardiometabolic health and colorectal health, and that the carbohydrate quality (i.e. a high intake of whole grains, legumes, vegetables and fruits and low intake of refined grains and sugary foods and beverages) is more important than quantity. In addition, there were no consistent benefits on clinical outcomes when changing the GI of a diet and that the findings from prospective studies of diets characterized by GI or GL are inconsistent.

There is evidence for a positive relationship between the intake of added or free sugars and risk of developing chronic metabolic diseases and dental caries. The EFSA Panel concludes that available data do not allow setting an upper level of intake for added or free sugars. Based on the risk of developing chronic metabolic diseases and on dental caries risk, the EFSA Panel considers that the intake of added and free sugars should be as low as possible in the context of a nutritionally adequate diet. However, the relationship between the consumption of added and free sugars at levels of intake below 10 E% and risk of chronic metabolic diseases could not be adequately explored owing to the low number of RCTs available, and therefore the uncertainty about the shape and direction of the relationships at these levels of intake is high.

The WHO finds that increasing or decreasing free sugars is associated with parallel changes in body weight. The relationship is present regardless of the level of intake of free sugars with the excess body weight associated with free sugars intake results from excess energy intake. The WHO recommends limiting free sugars to less than 10 E% (strong recommendation) based on moderate-quality evidence regarding body weight and dental caries and suggests a further reduction of the intake of free sugars to below 5 E% (conditional recommendation) based on very-low-quality evidence from ecological studies showing a dose-response relationship between intake of free sugars and dental caries. The conditional recommendation is set because there is less certainty about the balance between benefits and disadvantages of implementing this recommendation; however, no evidence was identified for harm associated with reducing the intake of free sugar to less than 5 E% (4). SACN recommended that the average population intake of free sugars should not exceed 5% of total dietary energy for age groups from 2 years upwards. In the Dietary Guidelines for Americans 2020–2025, the recommendation is to limit intakes of added sugars to less than 10 E% (3, 35), which is similar as in the 2015–2020 US Dietary Guidelines (36).

### Research gap

The numbers of studies in children on the relation between carbohydrate intake and health outcomes are scarce, as are studies in elderly. The main body of evidence among children relates to effects of added and free sugars on body weight/obesity and the effect of sugars exposure on lifelong dental caries, showing some evidence of negative effects. There is a lack of studies on carbohydrates and health effects in pregnancy health and outcomes, although some evidence exists on sugar-sweetened beverages and GDM and birthweight. There is a lack of long-term studies measuring the impact of reducing intake of added and free sugars (especially below 10 E%) on chronic metabolic diseases and surrogates. There are limitations with observational studies, including residual confounding and difficulties measuring habitual dietary intakes. There is a need for further development and use of objective biomarkers of carbohydrate quality (including added or free sugars and GI or GL). The evidence regarding adverse health effects is strong for sugar-sweetened beverages. However, there is a lack of studies (both RCTs and observational studies) examining the effects of other food sources of sugar (e.g. sweets, sweet bakery products, ice cream and sugary cereals). This is important because the main sources of added sugars in the Nordic countries are not beverages, but sweets, confectionery and sweet bakery products (37). There is a lack of standardized definition for dietary sugars (added and free sugars). A standardized definition would facilitate the comparison of results between studies. Future work will be necessary to identify cut-offs of added and free sugars.

## References

[1] Nordic Council of Ministers. Nordic Nutrition Recommendations 2012 – intergrating nutrition and physical activity. 5th ed. Copenhagen: Nordic Council of Ministres; 2014.

[2] Trumbo P, Schlicker S, Yates AA, Poos M. Dietary reference intakes for energy, carbohydrate, fiber, fat, fatty acids, cholesterol, protein and amino acids. J Am Diet Assoc 2002; 102(11): 1621–30. doi: 10.1016/S0002-8223(02)90346-9 12449285

[3] Dietary Guidelines Advisory Committee. Scientific Report of the 2020 Dietary Guidelines Advisory Committee: Advisory Report to the Secretary of Agriculture and the Secretary of Health and Human Services. Washington, DC: U.S. Department of Agriculture, Agricultural Research Service; 2020.

[4] WHO. Guideline: sugars intake for adults and children. WHO Guidelines Approved by the Guidelines Review Committee. Geneva: World Health Organization; 2015. 25905159

[5] Scientific Advisory Committee on Nutrition (SACN). Carbohydrates and Health. London: Public Health England; 2015.

[6] EFSA Panel on Nutrition NFaFA. Scientific opinion on the tolerable upper intake level for dietary sugars. EFSA J 2022; 20(2): 337. 10.2903/j.efsa.2022.7074 PMC8884083 35251356

[7] Sonestedt E, Overby NC, Laaksonen DE, Birgisdottir BE. Does high sugar consumption exacerbate cardiometabolic risk factors and increase the risk of type 2 diabetes and cardiovascular disease? Food Nutr Res 2012; 56: 1–19. doi: 10.3402/fnr.v56i0.19104 PMC3409338 22855643

[8] Blomhoff R, Andersen R, Arnesen EK, Christensen JJ, Eneroth H, Erkkola M, et al. Nordic Nutrition Recommendations 2023. Copenhagen: Nordic Council of Ministers; 2023. doi: 10.6027/nord2023-003

[9] Christensen JJ, Arnesen EK, Andersen R, Eneroth H, Erkkola M, Hoyer A, et al. The Nordic Nutrition Recommendations 2022 – principles and methodologies. Food Nutr Res 2020; 64: 1–15. doi: 10.29219/fnr.v64.4402 PMC7307430 32612489

[10] Höyer A, Christensen JJ, Arnesen EK. The Nordic Nutrition Recommendations 2022 – prioritisation of topics for de novo systematic reviews. Food Nutr Res 2021; 65: 7828. doi: 10.29219/fnr.v65.7828 PMC8897982 35291553

[11] Arnesen EK, Christensen JJ, Andersen R, Eneroth H, Erkkola M, Hoyer A, et al. The Nordic Nutrition Recommendations 2022 – structure and rationale of qualified systematic reviews. Food Nutr Res 2020; 64: 1–11. doi: 10.29219/fnr.v64.4403 PMC7307429 32612488

[12] European Food Safety Authority. Scientific opinion on dietary reference values for carbohydrates and dietary fiber. EFSA J 2010; 8: 1462. doi: 10.2903/j.efsa.2010.1462

[13] Reynolds A, Mann J, Cummings J, Winter N, Mete E, Te Morenga L. Carbohydrate quality and human health: a series of systematic reviews and meta-analyses. Lancet 2019; 393(10170): 434–45. doi: 10.1016/S0140-6736(18)31809-9 30638909

[14] Te Morenga L, Mallard S, Mann J. Dietary sugars and body weight: systematic review and meta-analyses of randomised controlled trials and cohort studies. BMJ 2013; 346: e7492. doi: 10.1136/bmj.e7492 23321486

[15] Moynihan PJ, Kelly SA. Effect on caries of restricting sugars intake: systematic review to inform WHO guidelines. J Dent Res 2014; 93(1): 8–18. doi: 10.1177/0022034513508954 24323509 PMC3872848

[16] World Cancer Research Fund, American Institute for Cancer Research. Diet, nutrition, physical activity and cancer: a global perspective. Continuous update project expert report. 2018.

[17] Jenkins DJ, Wolever TM, Taylor RH, Barker H, Fielden H, Baldwin JM, et al. Glycemic index of foods: a physiological basis for carbohydrate exchange. Am J Clin Nutr 1981; 34(3): 362–6. doi: 10.1093/ajcn/34.3.362 6259925

[18] Atkinson FS, Brand-Miller JC, Foster-Powell K, Buyken AE, Goletzke J. International tables of glycemic index and glycemic load values 2021: a systematic review. Am J Clin Nutr 2021; 114(5): 1625–32. doi: 10.1093/ajcn/nqab233 34258626

[19] Bjorck I, Liljeberg H, Ostman E. Low glycaemic-index foods. Br J Nutr 2000; 83 Suppl 1: S149–55. doi: 10.1017/S0007114500001094 10889806

[20] Salmeron J, Ascherio A, Rimm EB, Colditz GA, Spiegelman D, Jenkins DJ, et al. Dietary fiber, glycemic load, and risk of NIDDM in men. Diabetes Care 1997; 20(4): 545–50. doi: 10.2337/diacare.20.4.545 9096978

[21] Lemming EW, Pitsi T. The Nordic Nutrition Recommendations 2022 – food consumption and nutrient intake in the adult population of the Nordic and Baltic countries. Food Nutr Res 2022; 66: 1–11. doi: 10.29219/fnr.v66.8572 PMC9199833 35757440

[22] Jepsen S, Blanco J, Buchalla W, Carvalho JC, Dietrich T, Dorfer C, et al. Prevention and control of dental caries and periodontal diseases at individual and population level: consensus report of group 3 of joint EFP/ORCA workshop on the boundaries between caries and periodontal diseases. J Clin Periodontol 2017; 44 Suppl 18: S85–93. doi: 10.1111/jcpe.12687 28266120

[23] Louie JC, Tapsell LC. Association between intake of total vs added sugar on diet quality: a systematic review. Nutr Rev 2015; 73(12): 837–57. doi: 10.1093/nutrit/nuv044 26449366

[24] Tammi R, Maukonen M, Mannisto S, Sares-Jaske L, Kanerva N, Kaartinen NE. Association between added sugar intake and overall diet quality in the Finnish adult population. Br J Nutr. 2022; 128(9): 1848–1856. doi: 10.1017/S0007114521004736 34842126

[25] Mumena WA. Consumption of free sugar predicts nutrient intake of Saudi Children. Front Nutr 2021; 8: 782853. doi: 10.3389/fnut.2021.782853 34869544 PMC8634584

[26] Fujiwara A, Okada E, Okada C, Matsumoto M, Takimoto H. Association between free sugars intake and nutrient dilution among Japanese adults: the 2016 National Health and Nutrition Survey, Japan. Eur J Nutr 2020; 59(8): 3827–39. doi: 10.1007/s00394-020-02213-4 32162042

[27] Lai HT, Hutchinson J, Evans CEL. Non-milk extrinsic sugars intake and food and nutrient consumption patterns among adolescents in the UK national diet and nutrition survey, years 2008–16. Nutrients 2019; 11(7): 1–18. doi: 10.3390/nu11071621 PMC6682974 31319451

[28] Dello Russo M, Ahrens W, De Henauw S, Eiben G, Hebestreit A, Kourides Y, et al. The impact of adding sugars to milk and fruit on adiposity and diet quality in children: a cross-sectional and longitudinal analysis of the identification and prevention of dietary- and lifestyle-induced health effects in children and infants (IDEFICS) Study. Nutrients 2018; 10(10): 1–12. doi: 10.3390/nu10101350 PMC6213151 30248889

[29] Mok A, Ahmad R, Rangan A, Louie JCY. Intake of free sugars and micronutrient dilution in Australian adults. Am J Clin Nutr 2018; 107(1): 94–104. doi: 10.1093/ajcn/nqx008 29381794

[30] Jyvakorpi SK, Pitkala KH, Puranen TM, Bjorkman MP, Kautiainen H, Strandberg TE, et al. High intake of nonmilk extrinsic sugars is associated with protein and micronutrient dilution in home-dwelling and institutionalized older people. J Am Med Dir Assoc 2017; 18(4): 301–5. doi: 10.1016/j.jamda.2016.09.023 27887891

[31] Gibson S, Francis L, Newens K, Livingstone B. Associations between free sugars and nutrient intakes among children and adolescents in the UK. Br J Nutr 2016; 116(7): 1265–74. doi: 10.1017/S0007114516003184 27641637

[32] Moshtaghian H, Louie JC, Charlton KE, Probst YC, Gopinath B, Mitchell P, et al. Added sugar intake that exceeds current recommendations is associated with nutrient dilution in older Australians. Nutrition 2016; 32(9): 937–42. doi: 10.1016/j.nut.2016.02.004 27155956

[33] Sluik D, van Lee L, Engelen AI, Feskens EJ. Total, free, and added sugar consumption and adherence to guidelines: the dutch national food consumption survey 2007–2010. Nutrients 2016; 8(2): 70. doi: 10.3390/nu8020070 26828518 PMC4772034

[34] Gonzalez-Padilla E, J AD, Ramne S, Olsson K, Nalsen C, Sonestedt E. Association between added sugar intake and micronutrient dilution: a cross-sectional study in two adult Swedish populations. Nutr Metab 2020; 17: 15. doi: 10.1186/s12986-020-0428-6 PMC7011604 32071610

[35] U.S. Department of Agriculture and U.S. Department of Health and Human Services. Dietary Guidelines for Americans, 2020–2025. 9th Edition. December 2020. Available at DietaryGuidelines.gov.

[36] United States. Department of Health and Human Services, United States. Department of Agriculture, United States. Dietary Guidelines Advisory Committee. Dietary guidelines for Americans, 2015–2020. Eighth edition. ed. Washington, DC: U.S. Department of Health and Human Services and U.S. Department of Agriculture; 2015, 122p.

[37] EFSA Panel on Nutrition NFaFA. Annex D (Results of the intake assessment whole population) in Scientific opinion on the tolerable upper intake level for dietary sugars. EFSA J 2022; 20(2): Annex D. 10.2903/j.efsa.2022.7074 PMC8884083 35251356

